# MMP-13 *In-Vivo* Molecular Imaging Reveals Early Expression in Lung Adenocarcinoma

**DOI:** 10.1371/journal.pone.0132960

**Published:** 2015-07-20

**Authors:** Mathieu Salaün, Jing Peng, Harvey H. Hensley, Navid Roder, Douglas B. Flieder, Solène Houlle-Crépin, Olivia Abramovici-Roels, Jean-Christophe Sabourin, Luc Thiberville, Margie L. Clapper

**Affiliations:** 1 Cancer Prevention and Control Program, Fox Chase Cancer Center, Philadelphia, Pennsylvania, United States of America; 2 Laboratoire Quant.I.F – LITIS, EA 4108, Rouen University, Rouen, France; 3 Clinique Pneumologique & CIC INSERM U1404, Rouen University Hospital, Rouen, France; 4 Biological Imaging Facility, Fox Chase Cancer Center, Philadelphia, Pennsylvania, United States of America; 5 Department of Pathology, Fox Chase Cancer Center, Philadelphia, Pennsylvania, United States of America; 6 Department of Pathology, Rouen University Hospital, Rouen, France; National Cancer Institute, UNITED STATES

## Abstract

**Introduction:**

Several matrix metalloproteinases (MMPs) are overexpressed in lung cancer and may serve as potential targets for the development of bioactivable probes for molecular imaging.

**Objective:**

To characterize and monitor the activity of MMPs during the progression of lung adenocarcinoma.

**Methods:**

K-ras^LSL-G12D^ mice were imaged serially during the development of adenocarcinomas using fluorescence molecular tomography (FMT) and a probe specific for MMP-2, -3, -9 and -13. Lung tumors were identified using FMT and MRI co-registration, and the probe concentration in each tumor was assessed at each time-point. The expression of *Mmp2*, *-3*, *-9*, *-13 * was quantified by qRT-PCR using RNA isolated from microdissected tumor cells. Immunohistochemical staining of overexpressed MMPs in animals was assessed on human lung tumors.

**Results:**

In mice, 7 adenomas and 5 adenocarcinomas showed an increase in fluorescent signal on successive FMT scans, starting between weeks 4 and 8. qRT-PCR assays revealed significant overexpression of only *Mmp-13* in mice lung tumors. In human tumors, a high MMP-13 immunostaining index was found in tumor cells from invasive lesions (24/27), but in none of the non-invasive (0/4) (p=0.001).

**Conclusion:**

MMP-13 is detected in early pulmonary invasive adenocarcinomas and may be a potential target for molecular imaging of lung cancer.

## Introduction

Lung cancer is the leading cause of cancer-related death in the US, with a 5-year survival rate less than 20%. Because the poor prognosis of this disease is due in part to its late diagnosis, finding accurate tools for the early detection of lung cancer is of tremendous importance. Although CT screening studies have been effective in reducing lung-cancer mortality in high-risk patients [1], clinicians are now faced with the characterization of numerous peripheral lung nodules; more than 90% of which are benign [1]. This dilemma speaks clearly to the need for both an enhanced understanding of the biological basis of lung cancer and the identification of molecular targets for early detection and preventive intervention.

Adenocarcinoma is the most common histologic subtype of lung cancer and frequently carries a *KRAS* mutation (10–30% of cases). Mutation of *KRAS* is an early event in adenocarcinoma development, as it is found in atypical adenomatous hyperplasias and bronchoalveolar carcinomas [2]. The murine K-ras^LSL-G12D^ lung cancer model provides a way to study the development of lung tumorigenesis, from premalignant lesions to invasive cancer [3]. This mouse carries a point mutation at codon 12 of the *Kras* gene, and intratracheal delivery of Adenoviral Cre recombinase leads to removal of a transcriptional STOP element and induction of the expression of oncogenic K-ras specifically within the lungs. In this model, which has been validated as a good model of human adenocarcinoma, as it presents similar gene expression profile [4], lung atypical adenomatous hyperplasias (AAH), can be observed as soon as 4 weeks after infection, with adenomas and adenocarcinomas observed subsequently, 8 and 16 weeks postinfection, respectively [3].

The matrix metalloproteinases (MMPs) are a family of zinc-dependent proteins that degrade components of the extracellular matrix and play a major role in tumor invasion and metastasis [5]. MMPs are being explored as potential biomarkers, both diagnostic and prognostic, for a wide range of cancers. The molecular imaging of MMPs activity in cancer has successfully been applied to the detection of colorectal adenomas and ovarian epithelial cancer in mice [6, 7]. In humans, over expression of MMP-2, -7 and -9 in both early and late stages of lung cancer indicates that imaging these proteins may be useful in detecting and monitoring lung cancer progression [8, 9].

The goal of present study is to characterize the expression of MMPs throughout lung tumor development (AAHs, adenomas and adenocarcinomas). Use of an MMP-specific fluorescent probe and a dual MRI-FMT imaging system in the K-ras^LSL-G12D^ mouse model to detect and quantify tumor-associated activity in living mice led to the identification of MMP-13 expression as a potential marker of progression to lung adenocarcinoma. This hypothesis was confirmed by analyses of human lung tumors. Immunohistochemical staining of human lung adenocarcinomas for MMP-13 showed the significant increase of MMP-13 expression in invasive and minimally invasive adenocarcinomas, as compared to non invasive lesions.

## Material & Methods

### Animals

129/SvJ wild-type and mutant K-ras mice were maintained under pathogen-free conditions in a temperature controlled room on a 12:12-h light:dark cycle, with free access to water and food. Mice (8 weeks old) were anesthetized with Ketamine (100 mg/kg, ip) and Xylazine (5–10 mg/kg, ip), and infected with AdenoCre virus by intratracheal instillation (75 μL; 2.44x10^9^ pfu) (Ad5CMVCre, Gene Transfer Vector Core, University of Iowa, Iowa City, IA, USA). All animal procedures were approved by the Institutional Animal Care and Use Committee at Fox Chase Cancer Center.

### Fluorescence reflectance imaging

Initial experiments were carried out using an IVIS Spectrum (PerkinElmer) fluorescent/bioluminescent imaging system in order to ensure activation of the MMPSense680 probe in lung tumors. The thorax and abdomen of the mice were shaved 24h before imaging, and the mice were injected i.v with MMPSense680 (2 nmol). The animals were anesthetized continuously by isoflurane inhalation and fluorescent images were acquired in vivo with 0.2 second exposure times at f/stop = 2, with an excitation wavelength of 640 nm and emission wavelength of 700 nm. Following euthanasia by CO2 inhalation, the lungs were dissected and the lobes collected separately and imaged.

*Ex-vivo* tissue processing and histological analysis methods are described in the supplemental materials.

### Fluorescence Molecular Tomography Imaging

Prior to imaging, mice were anesthetized with 2% isoflurane (Isosol; Vedco, Inc, St Joseph, MO, USA) in medical-grade O_2_ and shaved. Twenty-four hours prior to imaging, animals were injected in the tail vein with 2 nmol MMPSense 680 (excitation wavelength = 680 ± 10 nm; emission wavelength 700 ± 10 nm) (PerkinElmer, Waltham, MA, USA). This bioactivable probe is optically silent until digestion by key matrix metalloproteinases (MMPs) including MMP-2, -3, -9 and -13, as described by the probe manufacturer, releasing the fluorophore from the quencher.

Animals were imaged at 4, 8, 12, 16, and 20 weeks after AdenoCre infection using the FMT 2000 Quantitative Tomography System (PerkinElmer, Waltham, MA, USA). Mice were anesthetized continuously by isoflurane inhalation and placed in a biplanar FMT imaging cassette. Careful alignment of the animal with markers on the imaging cassette allowed accurate placement of the regions of interest (ROIs) on successive scans during subsequent imaging analyses. The imaging cassette was transilluminated with laser light in the 680 nm channel of the FMT system. Resulting transmission and fluorescence patterns were captured with a thermoelectrically cooled CCD camera, and the position and intensity of fluorescence sources were reconstructed in three dimensions using the TrueQuant software package (Perkin Elmer), supplied with the FMT2000 [10].

During the last imaging session, the animal was placed in a customized biplanar, optically-silent holder that was compatible with both the FMT and MRI imaging cassettes. Two fiducial markers (1 cm each) containing the FMT-validated probe AngioSense680 (PerkinElmer) and gadolinium-DTPA (Gd-DTPA) (1:100 diluted in PBS; Magnevist, Berlex Labs, Hamilton, NJ, USA) were placed on the neck and abdomen of the animal to ensure coregistration of FMT & MRI images during post-acquisition processing. MRI scans were performed immediately after FMT, without removing the animal from the holder. Imaging times totaled approximately 30 minutes for each mouse.

### Magnetic Resonance Imaging

Immediately before the FMT scan, the animal received an intraperitoneal injection of Gd-DTPA (diluted 1:10 in PBS). Immediately after the FMT scan, MRI was performed in a vertical-bore 7-T magnet with a Bruker DRX300 spectrometer (Bruker Biospin Corporation, Billerica, MA) using ParaVision 3.0 software (Bruker Biospin Corporation) as described previously [11, 12]. Briefly, the first localizer scan of the thoracic area consisted of a 2D spin echo pulse sequence in the axial orientation, 20 slices, 1.5mm slice width, 256×128 matrix, minimum TR = 600 msec, 1 average, for a total imaging time of 1 min. After completion of the first localizer, a second localizer was proscribed in a sagittal orientation. MRI thoracic scans were then acquired in axial and sagittal orientations. Data sets consisted of an interleaved multi-slice spin echo pulse sequence. Imaging parameters were: slice thickness = 0.75 mm, field of view = 2.56 cm, in-plane resolution of 0.1 mm, with 4 signal averages and 20–30 slices, and repetition time (TR) = 500–700 msec (minimized for a given number of slices), for a total scan time of approximately 20 minutes.

### Image fusion, processing & analysis

FMT and MRI data sets were fused using freely available A Medical Imaging Data Examiner (AMIDE) software [13] (http://amide.sourceforge.net/). TrueQuant software permitted exportation of the FMT data sets in DICOM format. MRI data sets were imported directly into AMIDE. Fusion of the two image data sets was done using the fiducial markers, and the background probe signal of the liver. The data sets were aligned and rotated until the two markers and the liver overlapped in three dimensions.

Lung tumors were identified on MRI images and the corresponding ROIs were drawn on the FMT scans. The absolute value of probe retention in the ROI was measured in picomoles using the TrueQuant software. The ROI was then reported on the previous FMT scans to ensure quantification of the probe concentration during the development of the tumor(s).

### Ex vivo tissue processing & histological analysis

After *ex-vivo* imaging, each lung lobe was embedded in optimal cutting temperature (OCT) compound, frozen on dry ice and stored at -80°C. Tissue sections (5-μm) were cut from the frozen blocks, stained with hematoxylin and eosin and reviewed by an experienced pathologist. Lesions were classified as: AAHs—alveoli lined by a uniform population of hypertrophic cuboidal cells; adenomas—uniform epithelial cell population with rounded nuclei (without mitoses), less than 5 mm in diameter; adenocarcinomas—display greater cytologic atypia, increased mitotic index, regional variation in growth pattern, more papillary structures, and a diameter greater than 5 mm. Invasion of vessels and large airways or pleura are also observed [14].

### MMP gene expression in lung tumors

Real-Time quantitative PCR analyses were performed on frozen lungs from K-ras^LSL-G12D^ mice and wild-type animals infected with AdenoCre virus. Neoplastic pulmonary epithelial cells and adjacent nonneoplastic epithelium (∼2000 cells each) were isolated from frozen tissue sections (5 um) by laser capture microdissection using the PixCell II system (Arcturus Bioscience, Inc.). Total RNA was extracted using PicoPure RNA Isolation Kit (Arcturus Biosciences, Inc.) and analyzed using the Agilent RNA 6000 Pico kit and 2100 Bioanalyzer (Agilent Technologies). Samples with intact 18S and 28S rRNA were reverse-transcribed using the High Capacity cDNA Reverse Transcription Kit (Life Technologies). The cDNA was pre-amplified then subjected to quantitative PCR using TaqMan Universal PCR MasterMix gene-specific primers purchased from Life Technologies (Applied Biosystems): *Mmp-2*: Mm00439506_m1; *Mmp-3*: Mm00440295_m1; *Mmp-9*: Mm00442991_m1; *Mmp-13*: Mm01168713_m1; *Hprt1*: Mm00446968_m1, according to the manufacturer’s instructions. Each sample was run in triplicate. Genomic DNA contamination was assessed by inclusion of a control RT-PCR reaction in which reverse transcriptase was omitted. Water was used as the no template negative control and Mouse Reference Total RNA (Clontech) was used as the positive control. Detection and quantification of amplification products were monitored using an ABI7900 Sequence Detection System (Life Technologies). For the purpose of signal normalization, analysis of the expression of the internal reference gene *Hprt1* was carried out in triplicate for each sample. The internal reference gene was selected based on our previous experience [15] and existing literature [16]. Relative quantitation was calculated using the comparative Ct method (ΔΔCt; Applied Biosystems Reference Manual, User Bulletin #2). Ct values for transcripts of both the housekeeping gene (*Hprt1*) and the *Mmp* genes were below 30 in most samples. *Mmp-13* transcripts were non detectable in 3 normal samples.

### Immunohistochemistry

Immunohistochemistry was performed on formalin-fixed paraffin-embedded sections from 31 human lung samples that were selected based on the IASLC/ATS/ERS international multidisciplinary classification of lung adenocarcinomas [17]. Specimens included 2 AAHs, 2 adenocarcinomas *in situ* (AIS), 11 minimally invasive adenocarcinomas (MIA), and 16 invasive adenocarcinomas (3 papillary, 4 solid, 6 mucinous and 3 lepidic). Sections were stained with a mouse monoclonal antibody specific for MMP-13 (1:20 diluted Clone VIIIA2, from Lab Vision Corp., CA). Secondary antibodies conjugated with horseradish peroxidase were detected using the UltraView Universal Detection kit (Ventana Medical Systems, Inc., Tucson, AZ, USA). Positive control was normal placenta, and negative control was normal lung. The staining intensity of the neoplastic epithelial cells and fibroblasts was quantified by a pathologist, using a scoring system described previously [18]: no staining: 0; light brown particle in cytoplasm: 1; moderate brown particle in cytoplasm: 2; and dark brown particle in cytoplasm: 3. The percentage of cells positive for immunostaining in the entire section was quantified microscopically and classified into four groups. 1: <25% positive cells; 2: 25% to 50% positive cells; 3: 51% to 75% positive cells and 4: >75% positive cells. The staining index (SI), defined as the product of the intensity and the percentage of positive staining, was used to define high (SI ≥ 6) or low (SI < 6) expression of MMP-13 [18].

### Statistical Analysis

Fluorescence intensity was measured in *ex-vivo* IVIS images, and the average fluorescence intensity of the lobe/liver ratio was compared between adenomas, adenocarcinomas, and normal lung lobes, using the Mann-Whitney test.

The Mann-Whitney test was used to compare the probe concentrations between groups at each time point, calculated with the TrueQuant software from the FMT images. A p value <0.05 was considered significant.

Variance stabilizing and normalizing transformations was applied to the qRT-PCR data as appropriate. The Wilcoxon signed rank test was used to compare transcript levels (ΔC_T_) between normal and tumor tissue. To account for multiple comparisons, the Bonferroni correction procedure (p_B_ = 1-(1-p)^1/n^ where *p* is the uncorrected value, *p*_*B*_ is the corrected value of *p*, and *n* is the number of comparisons) was applied, and a p value <0.01 was considered significant.

The staining index for MMP-13 in human samples was compared between non-invasive and invasive lesions using Fisher’s exact test. A p value <0.05 was considered statistically significant.

## Results

### *In vivo* and *ex vivo* imaging of MMP activity in the K-ras^LSL-G12D^ lung cancer model

**Fig 1:**
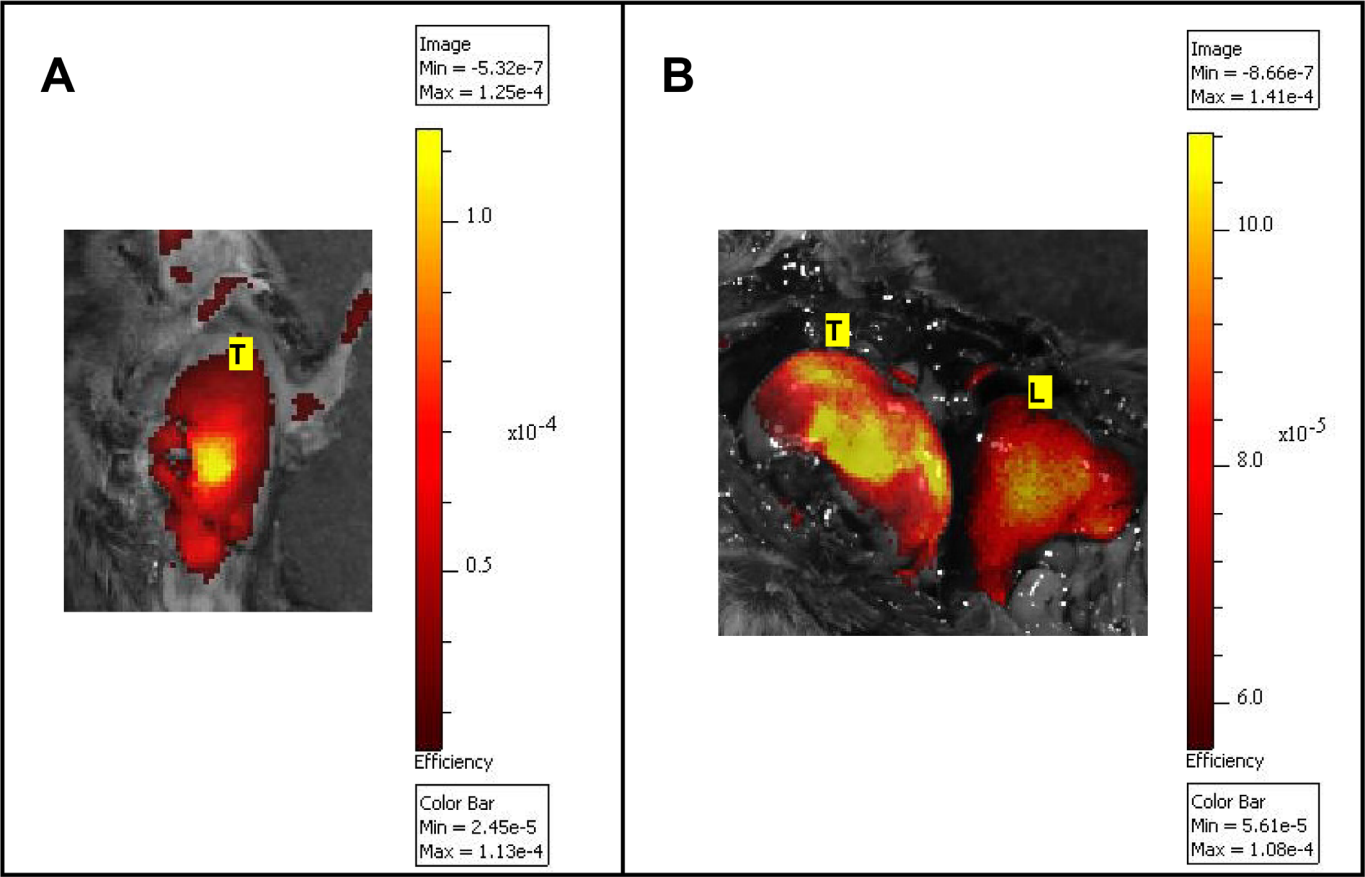
Imaging of MMP activity in the K-ras^LSL-G12D^ lung cancer model using MMPSense680. *In vivo* (A) and *ex vivo* (B) epi-fluorescent images (IVIS Spectrum) of a large superficial right lung tumor exposed after thoracotomy and laparotomy (B). The fluorescent bioactivatable probe reporting the proteolytic activity of MMP 2, -3, -9 and -13 (MMPSense680, 2 nmol) was injected i.v 24 h prior to imaging. The results confirmed the ability of an epi-illumination fluorescence system to image MMP activity in lung tumors, although the system could not discriminate large lung tumors from the liver (1A). Fluorescence intensity is represented as a pseudocolor image (MATLAB “hot” color map) overlaid on a white-light photographic image. Values of absolute fluorescence efficiency, as measured by the IVIS Spectrum, are shown on the color bar in units of 10^−5^ (1A) or 10^−4^ (1B). L: Liver; T: Right lung tumor.

**Fig 2:**
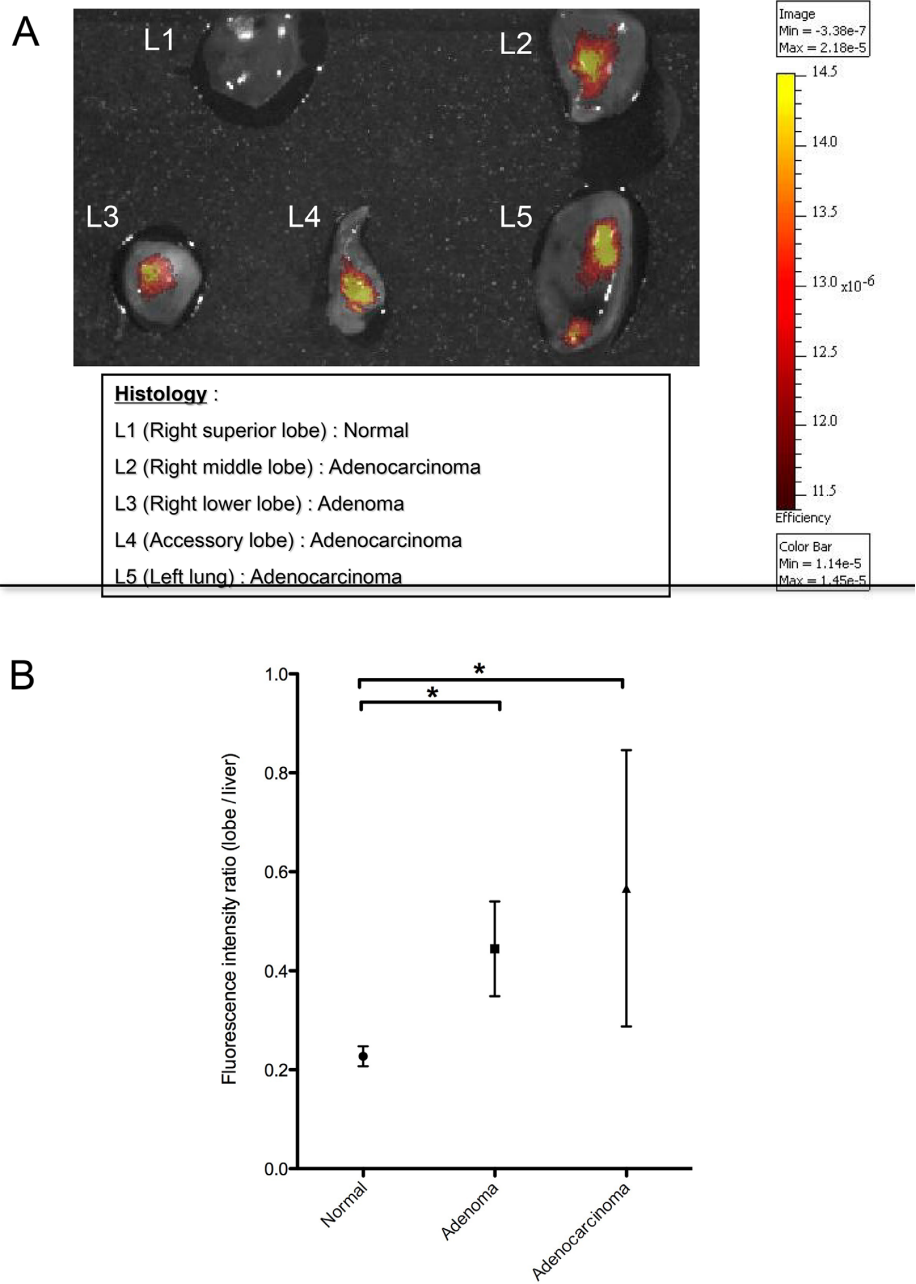
The fluorescent signal from MMPs increases in lung tumors. **A)** *Ex vivo* image of MMP activity in superficial lung lesions of various stages from K-ras^LSL-G12D^ mice. The nonneoplastic lobe (L1) of freshly isolated lungs imaged immediately after euthanasia did not display any fluorescent signal, while in the tumor-bearing lobes (L2-5), signal increased with the degree of lesion severity. The data confirmed the ability of an epi-illumination fluorescence system (IVIS Spectrum) to image MMP activity at different stages of lung tumor progression. **B)** Fluorescence intensity of lung adenomas (n = 8), adenocarcinomas (n = 3) and normal lung lobes (n = 25). In this experiment, the lung and tumor fluorescence signal was normalized on liver fluorescence, based on the hepatic activation of the probe. The fluorescence intensity ratio was calculated as a ratio of the median radiant efficiency of the lung lobe / liver. The median values ± interquartile range are shown, and the median differences between cancerous lesions and normal lobes are significant. * p value <0.05 by the Mann-Whitney test.

Initial experiments were performed using an epi-illumination fluorescence imaging system (IVIS Spectrum) to determine the feasibility of detecting MMP activity in lung tumors in the K-ras^LSL-G12D^ model. In this experiment, five K-ras^LSL-G12D^ mice (3 with 8 adenomas and 2 with 3 adenocarcinomas) and five wild-type control mice were instilled with the AdenoCre virus. Following injection of MMPSense680, mice were subjected to whole body imaging using the IVIS Spectrum. Only very large tumors (> 10 mm) were detectable *in vivo* with the epi-illumination imaging system (Fig 1). The technique was not sensitive enough to identify smaller lung tumors or specific lesions due to the lack of spatial resolution of deeply embedded tumors. However, tumors representative of all stages of the adenoma to carcinoma sequence were detected *ex vivo* (Fig 2). The fluorescence intensity increased significantly in adenomas and adenocarcinomas, as compared to normal lung tissue (Fig 2B). These experiments confirm the feasibility of imaging MMPs in lung tumors in the K-ras^LSL-G12D^ model, and demonstrate the reliability of the method to image the continuum for lung carcinogenesis.

### *In vivo* imaging of MMP expression during lung cancer progression

**Fig 3:**
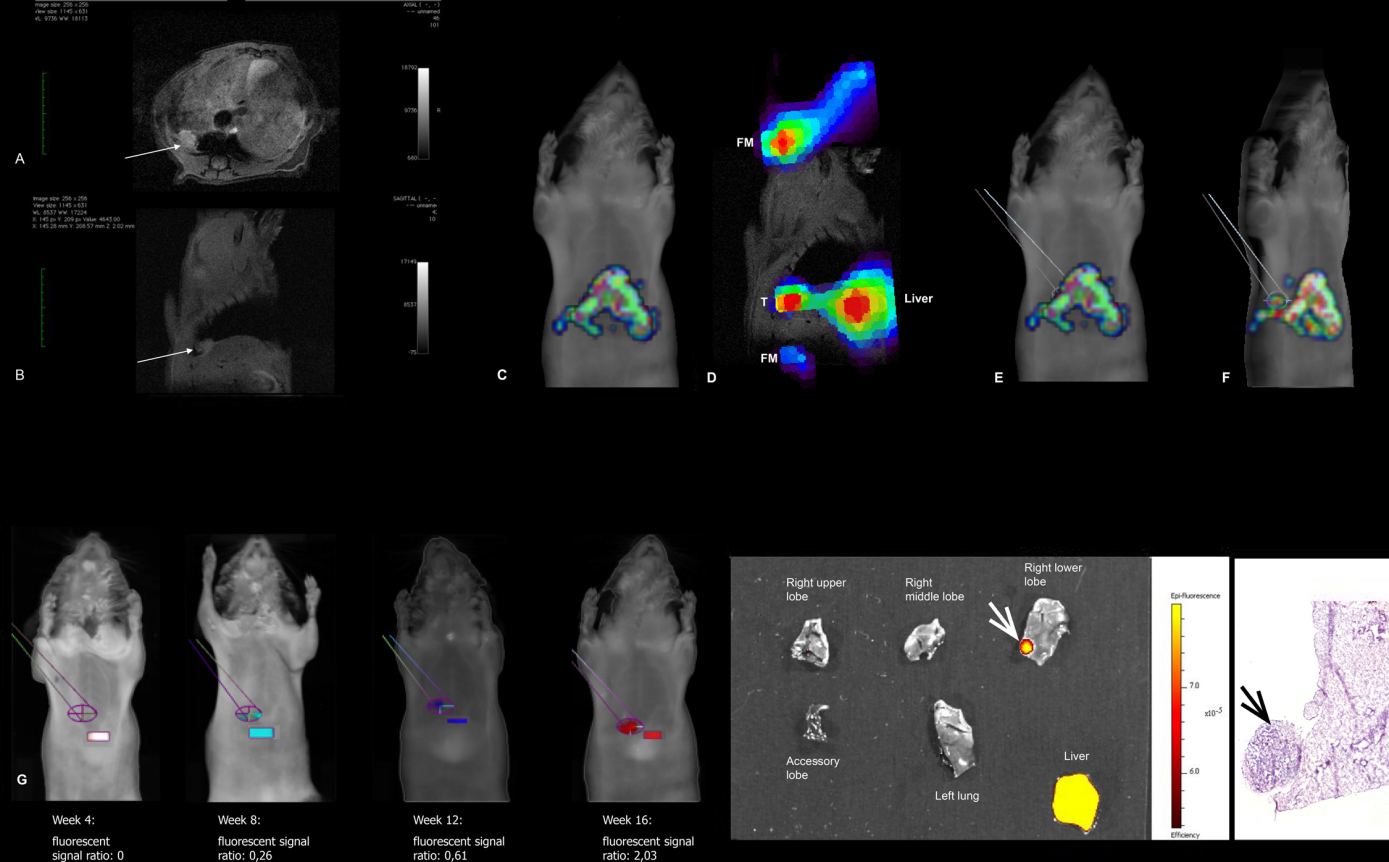
Retrospective analysis of the MMPSense680 fluorescent signal following identification of the tumor on fused FMT-MRI images. A representative example of one animal, from imaging to histology, is presented. **A&B)** Axial and sagittal MRI scans, showing a small tumor (white arrows) in the right lower lobe of the lung, behind the liver (L). **C)** Frontal view of the FMT scan. The tumor is hidden behind the strong fluorescent signal of the liver. **D)** Overlay of the FMT signal on the MRI image, using the fiducial markers (FM) and the liver (L) for 3D alignment. Note the distinct fluorescent signal localized to the tumor (T). **E&F)** The position of the tumor was identified on the FMT scan and the corresponding region of interest was circumscribed (E: frontal view of the FMT scan; F: oblique view of the same FMT scan. **G)** The tumor ROI (ellipse) was reported on the previous FMT scans of the same animal, along with a liver ROI (rectangle) to allow fluorescence ratio quantification, showing an increasing fluorescence with time. The effect of probe activation in the liver on measurements of this tumor—near the liver—cannot be measured. **H&I)** *Ex-vivo* epifluorescence imaging (H) and histological reviews (I) confirmed the presence of a 2mm adenoma in the right lower lobe (white and black arrows).

**Fig 4:**
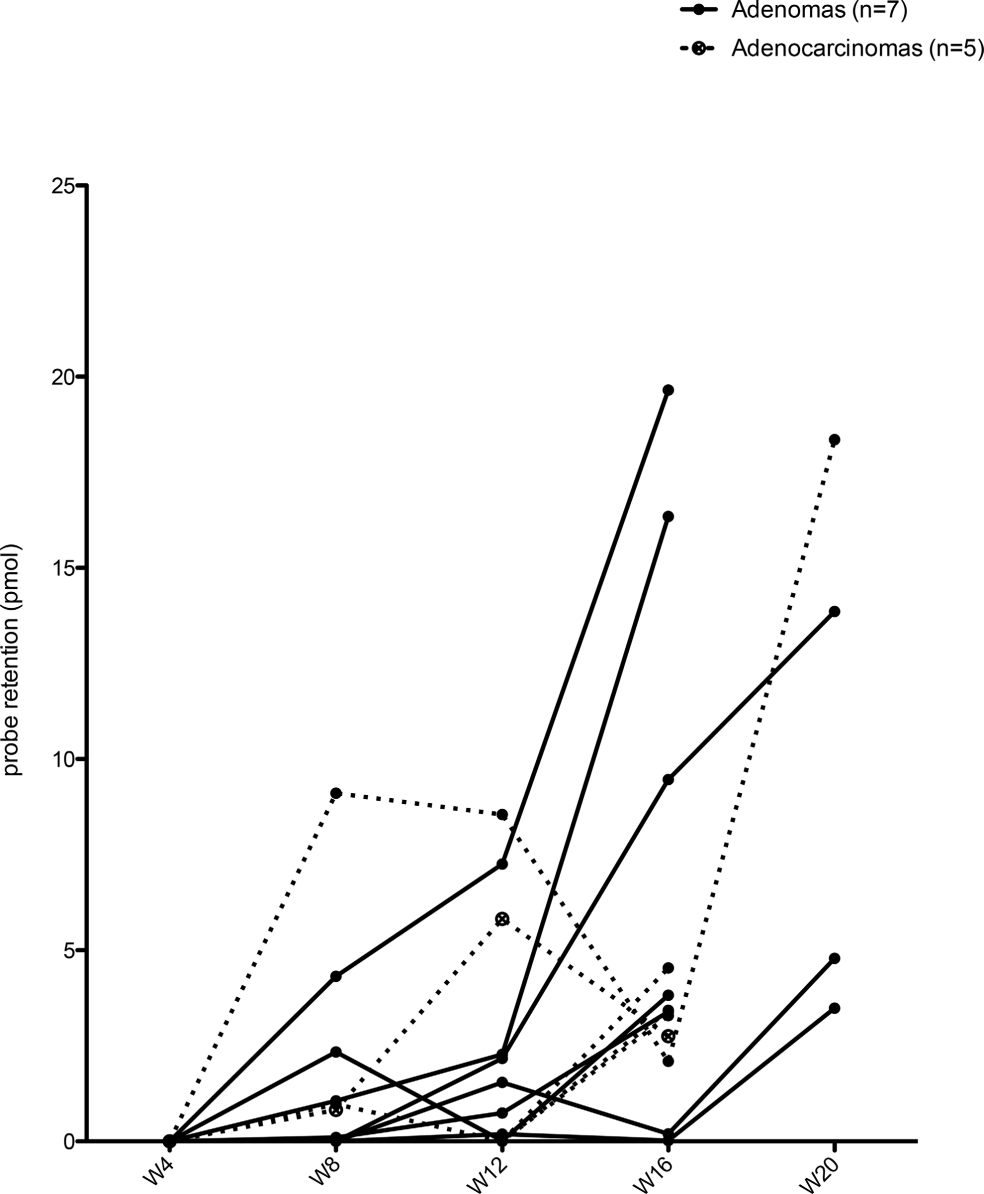
MMPSense680 retention was detected between week 4 and 8 in developing adenomas and adenocarcinomas and increased over time. Probe retention was calculated by the TrueQuant software in each ROI at each timepoint. Graph shows the probe retention in adenomas (n = 7) and adenocarcinomas (n = 5) at each time point.

Coupling of FMT with MRI provided a more sensitive method to detect MMP expression in lung tumors. This method allowed coregistration of volume and functional imaging and a way to monitor lesions longitudinally during tumor development and progression. The FMT-MRI fused images supported the identification of 12 lesions, 7 adenomas and 5 adenocarcinomas in 9 mice. To determine when MMPs expression was first detected, the lesions’ ROIs were tracked retrospectively on previous FMT scans, as shown in Fig 3. The retrospective follow-up of the lesions showed that the uptake of the fluorescent probe began between week 4 and 8. The intensity of the fluorescent signal increased over time (Fig 4). The effect of the probe activation in the liver on measurements on tumors near the liver cannot be measured.

### Relative RNA expression of MMPs in tumor *vs*. normal lung tissue in K-ras^LSL-G12D^ mice

**Fig 5:**
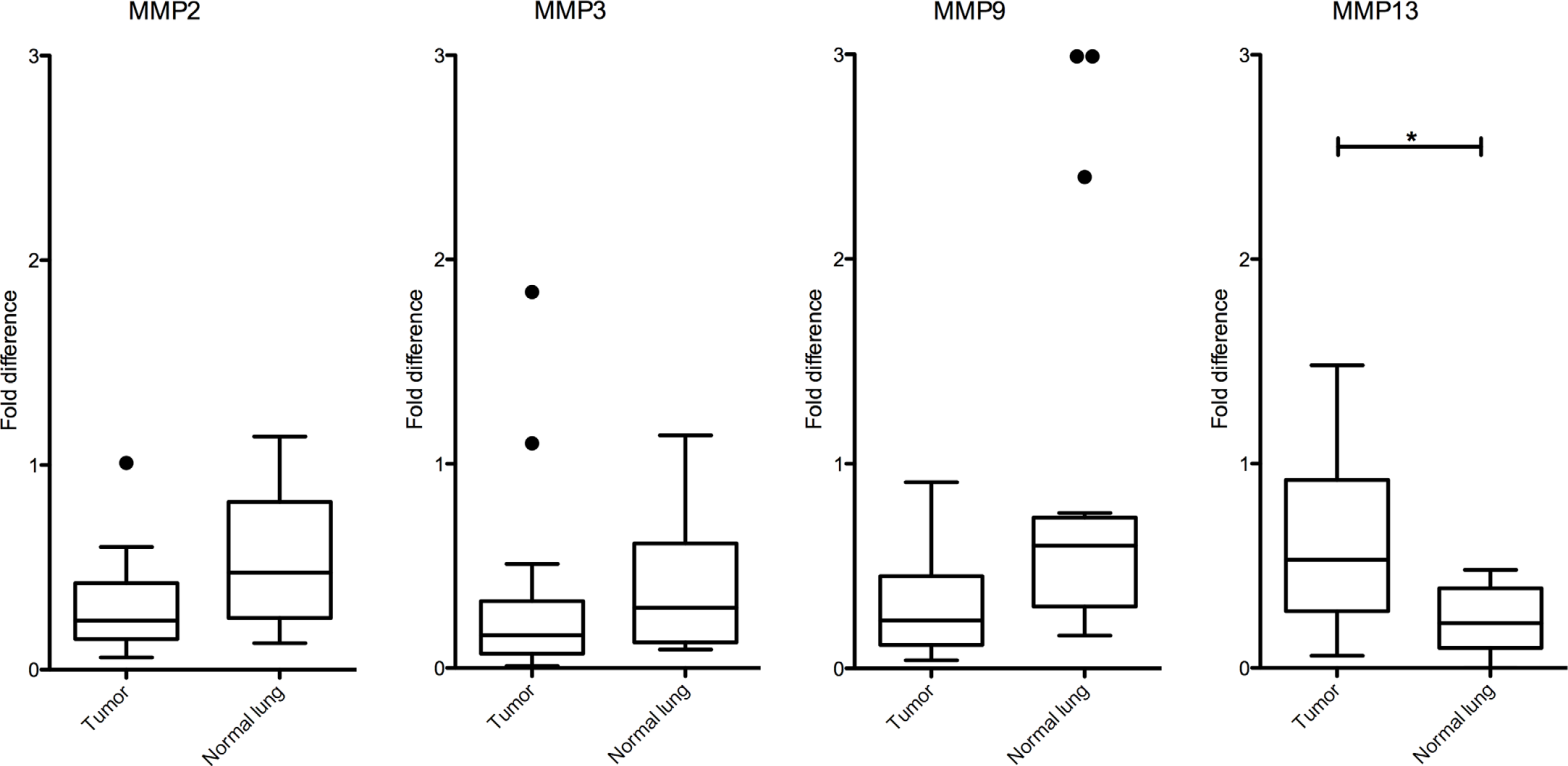
Relative RNA expression of *Mmp 2*, *-3*, *-9*, *-13* in tumor *vs*. normal lung tissue in K-ras^LSL-G12D^ mice. The expression of *Mmp-2*, *-3*, *-9*, and -*13* in lung lesions vs. normal lung tissue (n = 12 pairs) was compared using boxplots. The transcript level of each *Mmp* gene was determined by qRT-PCR and normalized against that of the housekeeping gene *Hprt*. The fold difference between each sample *vs*. a mouse universal reference total RNA was calculated using the delta delta Ct method. The middle line is the median, and the bottom & top of the box are the 25th and 75th percentiles (inter-quartile range, IQR). The "whiskers”, extending from the ends of the box, represent the most extreme point (≤ 1.5 times the IQR). More extreme points are considered outliers and are plotted separately. Only the relative expression of *Mmp-13* was significantly higher in cancerous tissue as compared to normal lung tissue (Wilcoxon signed rank test; * indicates a p value <0.01)

RT-qPCR was performed to assess the level of RNA expression of *Mmp-2*, *-3*, *9*, and -*13* in the 12 lesions compared to normal lung tissue. Only the relative expression of the *Mmp-13* gene was significantly higher in cancerous tissue as compared to normal lung tissue (median 2^-ddCT^ = 0.53 *vs*. 0.22 respectively; p = 0.006) (Fig 5). The level of expression of *Mmp-2* and *Mmp-3* was comparable in normal lung and tumor tissue (median 2^-ddCT^ = 0.24 *vs*. 0.47; p = 0.03 and 0.16 *vs*. 0.29; p = 0.28, respectively) whereas the relative expression of *Mmp-9* was significantly lower in cancerous tissue as compared to normal lung (median 2^-ddCT^ = 0.23 *vs*. 0.60; p = 0.008).

### MMP-13 expression in human lung adenocarcinomas

**Fig 6:**
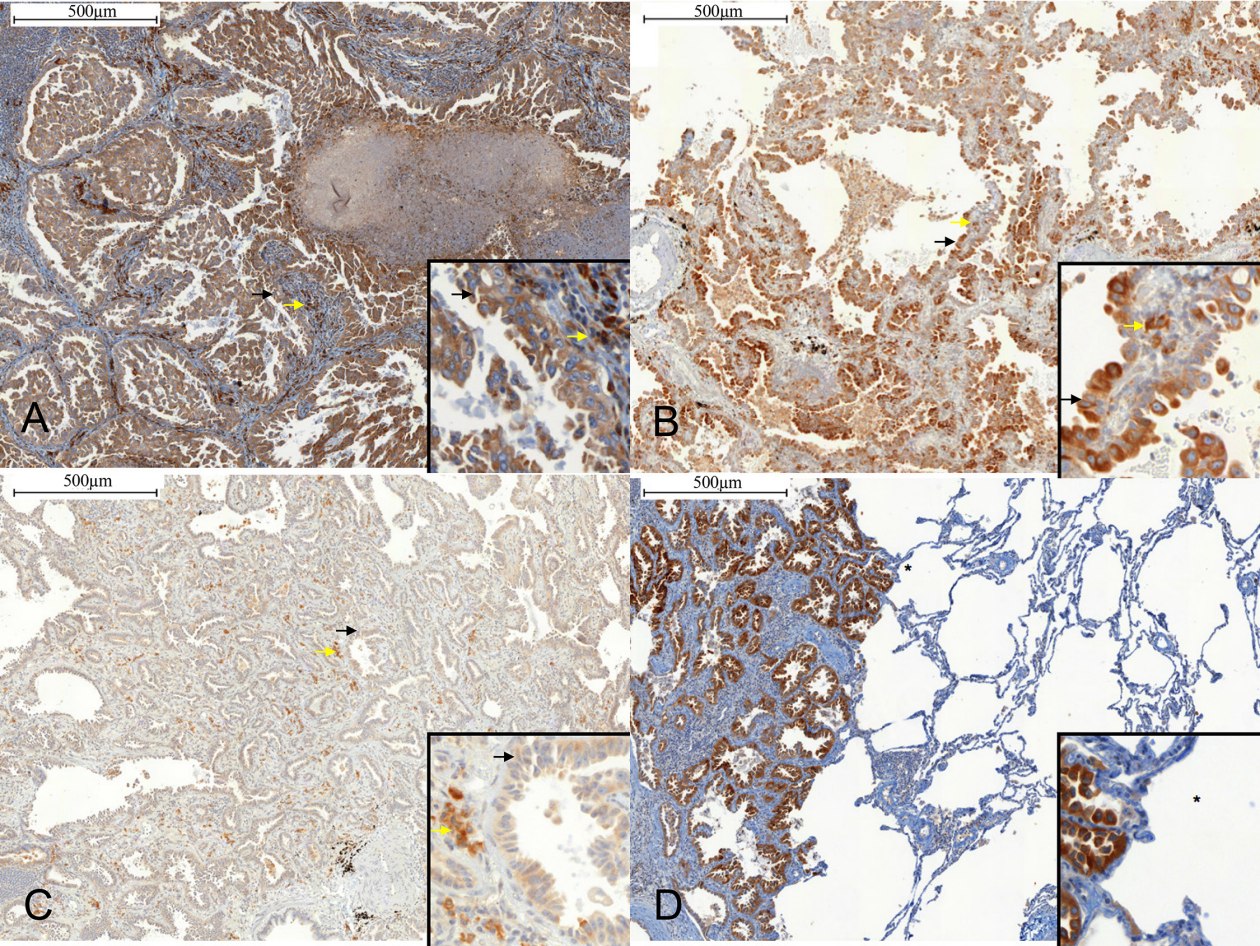
Expression of MMP-13 in human lung adenocarcinoma. Immunohistochemical staining was performed using Clone VIIIA2 diluted 1:20 (Lab Vision Corp., CA). **A)** Solid predominant adenocarcinoma. MMP-13 index staining (brown) is high in tumor cells (black arrow) and in fibroblasts (yallow arrow) (x100). **B)** Minimally invasive adenocarcinoma. MMP-13 staining is high in tumor cells (black arrow), and high in fibroblasts (yellow arrow) (x100). **C)** Adenocarcinoma *in-situ*. MMP-13 staining is high in tumor cells (black arrow), and high in fibroblasts (yellow arrow) (x100). **D)** Solid predominant adenocarcinoma and normal lung. Note the absence of MMP-13 staining in normal lung (*). Scale bar: 500 μm. Inserts in the x100 IHCs show x400 magnification.

Formalin-fixed paraffin-embedded sections of lung tissue from 31 patients diagnosed with pulmonary adenocarcinoma, including 4 non-invasive adenocarcinomas (2 atypical adenomatous hyperplasia (AAH), 2 adenocarcinoma *in situ* (AIS)), 11 minimally invasive adenocarcinomas (MIA), and 16 invasive lesions of different histological subtypes, were stained with antibodies against MMP-13 (Fig 6). The MMP-13 staining index for tumor cells was zero in the 2 AAHs and low in the 2 AISs, while it was high in 9 out of 11 MIAs and in 15 out of the 16 invasive lesions. Fibroblast staining was low in 1 of 2 AAHs, high in 2 of 2 AISs, low in 9 of 11 MIAs, and high in 9 of 16 invasive adenocarcinomas. A high MMP-13 staining index was significantly more frequent in tumor cells from invasive lesions (24/27) as compared to non-invasive lesions (0/4) (p = 0.001, Fisher’s exact test). No difference was found regarding MMP-13 expression in fibroblasts according to the severity of the lesions (p = 1; Fisher’s exact test). MMP13 staining was localized exclusively to the cytoplasm (Fig 6). Histological subtype, molecular analysis and MMP-13 staining index are detailed in Table 1. The MMP-13 staining index of *KRAS* mutated (n = 7) and *KRAS* wild-type (n = 7) tumors did not differ significantly; either tumor cells or fibroblasts (p = 1 for tumor cells and p = 1 for fibroblasts; Fisher’s exact test). Likewise, the MMP-13 staining index of *EGFR* mutated (n = 3) and *EGFR* wild-type (n = 22) tumors was comparable for both in tumor cells and fibroblasts (p = 1 for tumor cells and p = 0.23 for fibroblasts; Fisher’s exact test).

**Table 1:** Human pulmonary adenocarcinoma histological subtype, molecular analysis, and MMP-13 IHC staining index.

Patients	Histology	Molecular analysis	MMP-13 staining index		
Patient 1	Solid predominant adenocarcinoma	*EGFR*-WT		**Tumor cells**	**Fibroblasts**
	with mucin production		**% tumor cells**	4	1
			**Staining intensity**	3	3
			**Staining index**	**12**	**3**
Patient 2	Lepidic predominant invasive	*EGFR*-WT		**Tumor cells**	**Fibroblasts**
	adenocarcinoma		**% tumor cells**	4	1
			**Staining intensity**	2	3
			**Staining index**	**8**	**3**
Patient 3	Solid predominant invasive	*EGFR*-WT		**Tumor cells**	**Fibroblasts**
	adenocarcinoma with mucin	*KRAS* c.34G>T	**% tumor cells**	4	2
	production		**Staining intensity**	2	2
			**Staining index**	**8**	**4**
Patient 4	Solid predominant invasive	*EGFR*-WT		**Tumor cells**	**Fibroblasts**
	adenocarcinoma		**% tumor cells**	3	3
			**Staining intensity**	3	3
			**Staining index**	**9**	**9**
Patient 5	Solid predominant invasive	*EGFR*-WT		**Tumor cells**	**Fibroblasts**
	adenocarcinoma		**% tumor cells**	4	2
			**Staining intensity**	3	3
			**Staining index**	**12**	**6**
Patient 6	Solid predominant invasive	*EGFR*-WT		**Tumor cells**	**Fibroblasts**
	adenocarcinoma	*KRAS*-WT	**% tumor cells**	3	1
			**Staining intensity**	2	0
			**Staining index**	**6**	**0**
Patient 7	Solid predominant invasive	*EGFR* c.2573T>G		**Tumor cells**	**Fibroblasts**
	adenocarcinoma with lepidic		**% tumor cells**	3	1
	progression		**Staining intensity**	2	2
			**Staining index**	**6**	**2**
Patient 8	Minimally invasive	*EGFR*-WT		**Tumor cells**	**Fibroblasts**
	adenocarcinoma	*KRAS*-WT	**% tumor cells**	2	2
			**Staining intensity**	1	2
			**Staining index**	**2**	**4**
Patient 9	Papillary predominant invasive	*EGFR*-WT		**Tumor cells**	**Fibroblasts**
	adenocarcinoma		**% tumor cells**	4	3
			**Staining intensity**	2	3
			**Staining index**	**8**	**9**
Patient 10	Solid predominant invasive	*EGFR*-WT		**Tumor cells**	**Fibroblasts**
	adenocarcinoma + papillary		**% tumor cells**	4	2
			**Staining intensity**	3	3
			**Staining index**	**12**	**6**
Patient 11	Papillary predominant invasive	NA		**Tumor cells**	**Fibroblasts**
	adenocarcinoma		**% tumor cells**	1	1
			**Staining intensity**	2	2
			**Staining index**	**2**	**2**
Patient 12	Solid predominant invasive	NA		**Tumor cells**	**Fibroblasts**
	adenocarcinoma with mucin		**% tumor cells**	3	1
	production		**Staining intensity**	2	3
			**Staining index**	**6**	**3**
Patient 13	Solid predominant invasive	*EGFR*-WT		**Tumor cells**	**Fibroblasts**
	adenocarcinoma	*KRAS* c.35G>T	**% tumor cells**	4	3
			**Staining intensity**	3	3
			**Staining index**	**12**	**9**
Patient 14	Solid predominant invasive	*EGFR*-WT		**Tumor cells**	**Fibroblasts**
	adenocarcinoma with mucin		**% tumor cells**	4	2
	production		**Staining intensity**	3	3
			**Staining index**	**12**	**6**
Patient 15	Minimally invasive	*EGFR*-WT		**Tumor cells**	**Fibroblasts**
	adenocarcinoma	*KRAS* c.34_35delinsTT	**% tumor cells**	4	1
			**Staining intensity**	2	3
			**Staining index**	**8**	**3**
Patient 16	Solid predominant invasive	*EGFR*-WT		**Tumor cells**	**Fibroblasts**
	adenocarcinoma with mucin		**% tumor cells**	4	3
	production		**Staining intensity**	2	3
			**Staining index**	**8**	**9**
Patient 17	Minimally invasive	*EGFR*-WT		**Tumor cells**	**Fibroblasts**
	adenocarcinoma		**% tumor cells**	4	4
			**Staining intensity**	2	1
			**Staining index**	**8**	**4**
Patient 18	Solid predominant invasive	*EGFR*-WT		**Tumor cells**	**Fibroblasts**
	adenocarcinoma with lepidic		**% tumor cells**	3	4
	progression		**Staining intensity**	2	3
			**Staining index**	**6**	**12**
Patient 19	Minimally invasive	*EGFR* c.2297_2305dup		**Tumor cells**	**Fibroblasts**
	adenocarcinoma	*KRAS*-WT	**% tumor cells**	4	1
			**Staining intensity**	3	2
			**Staining index**	**12**	**2**
Patient 20	Solid predominant invasive	*EGFR*-WT		**Tumor cells**	**Fibroblasts**
	adenocarcinoma with mucin	*KRAS*-WT	**% tumor cells**	4	3
	production		**Staining intensity**	3	3
			**Staining index**	**12**	**9**
Patient 21	Atypical Adenomatous Hyperplasia	NA		**Tumor cells**	**Fibroblasts**
	(AAH)		**% tumor cells**	1	1
			**Staining intensity**	0	0
			**Staining index**	**0**	**0**
Patient 22	Atypical Adenomatous Hyperplasia	NA		**Tumor cells**	**Fibroblasts**
	(AAH)		**% tumor cells**	1	3
			**Staining intensity**	0	1
			**Staining index**	**0**	**3**
Patient 23	Minimally invasive	*EGFR*-WT		**Tumor cells**	**Fibroblasts**
	adenocarcinoma	*KRAS* c.37G>T	**% tumor cells**	1	1
			**Staining intensity**	1	2
			**Staining index**	**1**	**2**
Patient 24	Minimally invasive	*EGFR*-WT		**Tumor cells**	**Fibroblasts**
	adenocarcinoma	*KRAS*-WT	**% tumor cells**	4	2
			**Staining intensity**	2	2
			**Staining index**	**8**	**4**
Patient 25	Minimally invasive	*EGFR*-WT		**Tumor cells**	**Fibroblasts**
	adenocarcinoma	*KRAS* c.34G>C	**% tumor cells**	3	1
			**Staining intensity**	2	3
			**Staining index**	**6**	**3**
Patient 26	Minimally invasive	*EGFR* c.2573T>G		**Tumor cells**	**Fibroblasts**
	adenocarcinoma	*KRAS*-WT	**% tumor cells**	4	1
			**Staining intensity**	2	2
			**Staining index**	**8**	**2**
Patient 27	Minimally invasive	NA		**Tumor cells**	**Fibroblasts**
	adenocarcinoma		**% tumor cells**	4	3
			**Staining intensity**	3	3
			**Staining index**	**12**	**9**
Patient 28	Minimally invasive	NA		**Tumor cells**	**Fibroblasts**
	adenocarcinoma		**% tumor cells**	4	2
			**Staining intensity**	3	3
			**Staining index**	**12**	**6**
Patient 29	Minimally invasive	*EGFR*-WT		**Tumor cells**	**Fibroblasts**
	adenocarcinoma	*KRAS* c.34G>T	**% tumor cells**	4	1
			**Staining intensity**	3	1
			**Staining index**	**12**	**2**
Patient 30	Adenocarcinoma *in situ*	*EGFR*-WT		**Tumor cells**	**Fibroblasts**
		*KRAS* c.35G>A	**% tumor cells**	4	2
			**Staining intensity**	1	3
			**Staining index**	**4**	**6**
Patient 31	Adenocarcinoma *in situ*	*EGFR*-WT		**Tumor cells**	**Fibroblasts**
		*KRAS*-WT	**% tumor cells**	4	2
			**Staining intensity**	1	3
			**Staining index**	**4**	**6**

Abbreviations: MMP-13: matrix-metalloproteinase 13; *EGFR*: Epidermal Growth Factor Receptor; WT: Wild-type; *KRAS*: Kirsten rat sarcoma viral oncogene homolog; NA: Not available

Staining index scoring: % tumor cells positive for immunostaining: 4: >75% of tumor cells stained; 3: 50–75% of the tumor cells stained; 2: 25–50% of the tumor cells stained; 1: <25% of the tumor cells stained. Staining intensity: 0: no staining:; 1: light brown particle in cytoplasm; 2: moderate brown particle in cytoplasm; 3: dark brown particle in cytoplasm. The staining index (SI), defined as the product of the intensity and the percentage of positive staining, was used to define high (SI ≥ 6) or low (SI < 6) expression of MMP-13.

## Discussion

Data from the present study demonstrate that MMP-13 is expressed early during lung adenocarcinoma development. *In vivo* imaging of MMP activity in this murine model of step-by-step development of adenocarcinoma lead to the detection of MMP expression early at 4 weeks of cancer development. RT-qPCR identified *Mmp-13* as the main *Mmp* expressed in both in adenomas and carcinomas. MMP-13 was also remarkably expressed in minimally invasive and invasive lung adenocarcinoma in humans, independently of the mutation status.

Extracellular matrix degradation during tumor invasion and metastasis is largely mediated by MMPs (see ref. [19] for review). MMP-13 is able to cleave fibrillar collagens [20]. It was discovered in breast cancer [21] and has been described in many pathological conditions such as arthritis [22] or atherosclerosis [23]; its expression appears to be very low in normal tissue [21, 24]. Overexpression of MMP-13 has been reported for head and neck squamous carcinomas (HNSCCs) [25, 26], esophageal, gastric and colorectal cancers [27–29] and skin cancers [30]. In precancerous conditions, MMP-13 expression varies among target organs, being overexpressed in adenomatous colon polyps [31], but not present in premalignant lesions of the skin [30]. Moreover, in HNSCCs, urinary tract or skin carcinomas, MMP-13 expression is observed not only in tumor cells but also in stromal cells surrounding the tumor [25, 26, 32], in particular those located at the invading front [26, 30, 33]. This suggests that MMP-13 may play a key role in orchestrating the early signaling events required for cancer cell invasion.

While expression of MMP-2 and MMP-9 have been characterized extensively in lung cancer, much less is known about MMP-13 [9, 34]. Galateau-Salle et al. reported the role of MMP-2 and -9 in the successive steps of the invasion process during the development of squamous bronchial lesions [35]. Overexpression of several MMPs, including MMP-13, in non-small cell lung cancer (NSCLC) has been observed [36]. Thomas et al. reported that MMP-13, the most frequently expressed proteinase, was detected more frequently in adenocarcinomas as compared to squamous cell carcinomas. Data from other investigators suggest that expression of MMP-13 may serve as a biomarker of poor prognosis in NSCLC patients, associated with bone marrow microinvolvement by tumor cells [37]. To our knowledge, the expression of MMP-13 in early pulmonary adenocarcinomas, has not been reported previously.

Using gene expression profiling, the K-ras model used in this study has been validated as an accurate model of human pulmonary adenocarcinoma, as it reproduces the genetic alterations present in human tumors [4]. If our results show differences with the human adenocarcinomas regarding the expression MMP-2, -7 and -9, the overexpression of MMP-13 is consistent with human tumors. This model offers the opportunity to assess *in vivo* both the early and late stages of lung adenocarcinoma. Recently, the model allowed the longitudinal study of lung cancer progression using FMT and cathepsin-activated probes [38]. In the present study, this model was used to identify MMP-13 as a target for early detection and progression of lung cancer using FMT. Different patient-like orthotopic animal models of lung cancer with GFP-transfected cell lines could be used to track metastatic cells and study the influence of MMP-13 on the metastatic niche [39–41]. In such animal models, the use of transgenic mice expressing fluorescent proteins, and transplanted with lung cancer cell lines expressing another fluorescent protein, could also be used to study tumor-host interactions, with respect to the expression of different MMPs [41, 42]. Based on the current availability of fluorescent proteins of many different colors, “color-coded” cancer cells can be used to study the impact of different genotypes or phenotypes, and investigate various cellular functions including motility, invasion, angiogenesis and metastasis [43–46]. Simultaneous use of different fluorophores in the same subject would require spectral separation imaging to distinguish specific colors including autofluorescence [42].

As stated in the review paper on *Classification of Proliferative Pulmonary Lesions of the Mouse*: *Recommendations of the Mouse Models of Human Cancers Consortium*, by Nikitin *et al*. [14], the mouse classifications do not match those used in human pulmonary pathology. In contrast to the mouse models where adenomas are considered both a truly benign tumor or-as in the the K-ras^LSL-G12D^ lung cancer model- an intermediate in the adenoma-adenocarcinoma sequence, adenomas in humans represent benign lesions that will never progress to lung adenocarcinomas [14, 17, 47]. Therefore, we used immunohistochemical analyses of archived human lung tissue, including pre-cancerous and minimally invasive lesions, to confirm that MMP-13 is expressed during the early phases of adenocarcinoma development, including in minimally invasive lesions, but not in non-invasive lesions (AIS and AAH).

MMP-13 expression may be of clinical interest as, in the major CT screening study, more than 90% of the identified peripheral lung nodules were benign, and more than 70% required further follow-up procedures [1]. In this context, the development of fluorescent smart-probes specific for aggressive lesions would be of great interest. These probes may be usefully coupled with recent *in vivo* and *in situ* microimaging techniques such as probe-based confocal laser endomicroscopy (pCLE). pCLE is an endoscopic technique that has recently been applied to the exploration of distal lung diseases [48–50], and is currently evaluated to image distal lung nodules (ClinicalTrials.gov Identifier:NCT01931579). Pulmonary pCLE has already been evaluated with fluorescent smart probes, to image invasive pulmonary aspergillosis [51]. The use of pCLE associated to fluorescent smart probes or antibodies to image human digestive diseases *in vivo* has also been reported [52, 53]. Because of its role in early stage cancer development, and absence in normal tissue [21, 24], MMP-13 may also be considered in the future as a relevant target for molecular imaging of lung cancer *in vivo*.

In conclusion, this study highlights the importance of MMP-13 in lung adenocarcinoma development, and emphasizes its relevance as a target for innovative molecular imaging. Because it may differentiate invasive from non-invasive lung adenocarcinomas, these results may also have therapeutic implications in the future.
